# Relationship between multimorbidity, disease cluster and all-cause mortality among older adults: a retrospective cohort analysis

**DOI:** 10.1186/s12889-021-11108-w

**Published:** 2021-06-05

**Authors:** Kun He, Wenli Zhang, Xueqi Hu, Hao Zhao, Bingxin Guo, Zhan Shi, Xiaoyan Zhao, Chunyu Yin, Songhe Shi

**Affiliations:** 1 Department of Epidemiology and Health Statistics, College of Public Health, Zhengzhou University, 100 Kexue Avenue, Zhengzhou, Henan 450001 People’s Republic of China; 2 Department of Pharmacy, Zhengzhou People’s Hospital, Zhengzhou, Henan People’s Republic of China; 3 Department of Neurology, Chinese People’s Liberation Army General Hospital, Beijing, People’s Republic of China

**Keywords:** Older adults, Chronic conditions, Multimorbidity, Disease cluster, All-cause mortality

## Abstract

**Background:**

Previous studies have evaluated the association of multimorbidity with higher mortality, but epidemiologic data on the association between the disease clusters and all-cause mortality risk are rare. We aimed to examine the relationship between multimorbidity (number/ cluster) and all-cause mortality in Chinese older adults.

**Methods:**

We conducted a population-based study of 50,100 Chinese participants. Multiple logistic regression analysis was used to estimate the impact of long-term conditions (LTCs) on all-cause mortality.

**Results:**

The prevalence of multimorbidity was 31.35% and all-cause mortality was 8.01% (50,100 participants). In adjusted models, the odds ratios (ORs) and 95% confidence intervals (CIs) of all-cause mortality risk for those with 1, 2, and ≥ 3 LTCs compared with those with no LTCs was 1.45 (1.32–1.59), 1.72 (1.55–1.90), and 2.15 (1.85–2.50), respectively (*P*_trend_ < 0.001). In the LTCs ≥2 category, the cluster of chronic diseases that included hypertension, diabetes, CHD, COPD, and stroke had the greatest impact on mortality. In the stratified model by age and sex, absolute all-cause mortality was higher among the ≥75 age group with an increasing number of LTCs. However, the relative effect size of the increasing number of LTCs on higher mortality risk was larger among those < 75 years.

**Conclusions:**

The risk of all-cause mortality is increased with the number of multimorbidity among Chinese older adults, particularly disease clusters.

**Supplementary Information:**

The online version contains supplementary material available at 10.1186/s12889-021-11108-w.

## Background

Aging around the world poses a global challenge, previous studies have reported that thousands of persons turn 65 years old every day [1]. To our knowledge, China has the largest population base in the world, and the proportion of the elderly population is also increasing year by year. In 2009, the number of Chinese people aged 65 or above had reached 113,09 million, accounting for 8.5% of the total population. By 2019, the population aged 65 and above in China reached 176.03 million, accounting for 12.6% of the total population. In 10 years, the population increased by 55.65%, which means that China is aging at an unprecedented rate [2]. The phenomenon of ageing has led to a substantial increase in chronic conditions, which consequently results in a rising prevalence of multimorbidity, most commonly described as the presence of two or more long-term conditions (LTCs) [3]. Studies have found that multimorbidity leads to poor quality of life [4], increased use of inpatient and ambulatory greater health care [5, 6], greater complexity regarding of clinical treatment and patient management [7, 8], and more importantly, an increase in mortality [9, 10]. For example, previous studies, mainly based on the UK population, had suggested that the all-cause mortality rate for people with multimorbidity was 4.74% [11]. Thus, the management of multimorbidity has become a public health priority for public health care professionals and health care systems [7, 12].

The study found that hypertension, diabetes, stroke, heart disease, chronic respiratory disease and cancer are among the most common types of multimorbidity and are the most common causes of long-term disability and premature mortality worldwide [13–16]. As a result of the change of healthy lifestyle and the progress of medical level, the mortality rate from multimorbidity such as heart disease and cancer has been significantly reduced in high-income countries [17, 18], whereas rates of mortality caused by multimorbidity are rising rapidly in low- and middle-income countries, posing a serious social burden [19].

Thus, a better understanding of the epidemiology of multimorbidity is necessary to develop interventions to reduce the burden of death. However, the relationship between multimorbidity (number/cluster) and all-cause mortality has not been well described in the Chinese population. Therefore, we designed a retrospective cohort study to explore the relationship in Chinese older adults.

## Methods

### Study design and participants

The present study was based on an annual health examination dataset in the Xinzheng electronic health Management Center. The annual health examination program is part of the National Basic Public Health Service program, which is led and organized by Xinzheng Municipal Health Committee. Xinzheng CDC, township health centers and community health service centers of Xinzheng are responsible for the organization and implementation of the survey. From January 1, 2014 to November 15, 2019, a total of 84,353 (81.3%) participants were successfully followed up. We excluded participants with age < 65 at baseline (*n* = 31,900), or missing data for date of birth and gender at baseline (*n* = 2353). A total of 50,100 individuals were included in the main analysis (Supplemental File 1: Figure S1). The study was approved by the Ethics Committee of Zhengzhou University (Reference Number: ZZUIRB2019–019), and written informed consent was obtained from all participants. The research team obtained a license to use the data, which was granted by the Xinzheng Municipal Health Committee.

### Data collection

A standardized questionnaire was used by trained research staff under stringent quality control to collect information. Marital status was categorized as living with partner and without partner. Smokers were defined as ever smoking at least 100 cigarettes in their lifetime and classified as nonsmokers and previous/current smokers [20]. Alcohol consumption was divided into three categories: never, occasionally or daily. Physical activity was a categorical variable based on the self-reporting and classified as never, occasionally or daily. The measurements for height and weight were performed with the subjects wearing light clothing without shoes [21]. Blood pressure was measured by an automatic sphygmomanometer (Omron HEM-7125, Kyoto, Japan) after subjects had rested in a seated position for at least 5 min, and the mean value was recorded when the subjects were measured three times [22]. Blood samples were obtained after an overnight fast of at least 8 h to assess levels of fasting plasma glucose (FPG) using an automatic biochemical analyzer (DIRUI CS380, Changchun, China) [22]. In this study, diabetes was defined as fasting glucose levels ≥7.0 mmol/L and/or current treatment with anti-diabetes medication according to the China guideline for T2DM [23]. Hypertension was defined as positive for participants who were considered hypertensive with systolic blood pressure (SBP) ≥140 mmHg or diastolic blood pressure (DBP) ≥90 mmHg or current use of antihypertensive medication [24]. Body mass index (BMI) was calculated as weight (kg) divided by height squared (m^2^), which was classified based on the WHO classification criteria into underweight (< 18.5 kg/m^2^), desirable (18.5–24.9 kg/m^2^), overweight (25.0–29.9 kg/m^2^), obesity class I (30.0–34.9 kg/m^2^), obesity class II (35.0–39.9 kg/m^2^), and obesity class III (≥40.0 kg/m^2^) [25]. Information on the 7 chronic conditions diagnosed by a physician or reported by the study participants was recorded: (1) hypertension, (2) diabetes, (3) coronary heart disease (CHD), (4) stroke, (5) chronic obstructive pulmonary disease (COPD), (6) cancer, and (7) mental system disease. Multimorbidity was classified based on number of chronic conditions into no LTCs, 1 LTC, 2 LTCs, and ≥ 3 LTCs.

### Outcome definition

The main outcome was death from any cause as of November 15, 2019. The date and cause of death for the study subjects was obtained from the local CDC’s cause of death reporting system, and the cause of death was determined by medical records and death certificates. The International Classification of Diseases, 10th Edition (ICD-10) diagnostic codes were used to classify deaths as those caused by cardiovascular disease (ICD-10 codes I20-I25 and I60-I69), cancer (ICD-10 codes C00-C97) or other causes [26].

### Statistical analysis

All quantitative variables are described using the median (interquartile range [IQR]) for skewed distribution, and qualitative variables are expressed as frequency (%). Multiple logistic regression analysis was used to estimate odds ratios (ORs) and 95% confidence intervals (CIs) for risk of all-cause mortality by groups of LTC categories. With no LTCs as the reference group, we constructed three multivariate-adjusted logistic regression models: model 1 was adjusted for age, sex and marital status; model 2 was adjusted for model 1 plus smoking status, alcohol status and physical activity; and model 3 was further adjusted for BMI. In order to understand the pattern and effect of multimorbidity, we further analyzed the common disease cluster in older adults. A log likelihood ratio test for multiplicative interaction showed that age (< 75/≥75 years) at baseline modified the association of LTCs and risk of all-cause mortality (*p*-interaction < 0.05). For subgroup analyses, we stratified participants by age and sex at baseline. In addition, sensitivity analysis was performed in two cases: the first was in a population whose LTCs was defined by the medical examination diagnostic record at study baseline and not including self-reported history, and the second was included in the model analysis using BMI classification criteria for Asian adults [27].

Finally, to express the impact of multimorbidity on risk of all-cause mortality in the participants, we estimated the population attributable risk percent (PAR%) and the number needed to screened (NNBS). PAR% was estimated as (*I*_t_ - *I*_0_)/*I*_t_*100%, in which *I*_t_ is the mortality rate in the population and *I*_0_ is the mortality rate in the non-multimorbid group [28]. NNBS was estimated as 1/(*I*_e_ - *I*_0_), in which *I*_e_ is the mortality rate in the multimorbid group and *I*_0_ is the mortality rate in the non-multimorbid group [29].

Statistical analyses were performed using SAS 9.1 (SAS Institute, Cary, NC, USA), the forest plot was performed by GraphPad Prism 8, and the level of significance was considered at *P* < 0.05 (two-tailed).

## Results

The baseline characteristics of study participants with the four LTC groups are presented in Table 1. A total of 4012 (8.01%) of the 50,100 participants died. The median age at baseline was 69.18 years (range 65–106 years), and 53.87% of the population was female. The proportion of participants with 0, 1, 2, and 3 or more LTCs was 12,334 (24.62%), 22,060 (44.03%), 12,866 (25.68%), and 2840 (5.67%), respectively. In addition, the prevalence of multimorbidity in female was higher than in male (67.47% vs 63.58%). The most frequent diseases in participants with 3 or more LTCs were hypertension (98.87%), CHD (89.65%), and diabetes (88.59%).

**Table 1:** Relationship of multimorbidity with demographics and health-related behaviour at baseline

	No LTCs *N* = 12,334 (24.62%)	1 LTC *N* = 22,060 (44.03%)	2 LTCs *N* = 12,866 (25.68%)	≥3 LTCs *N* = 2840 (5.67%)	Over all *N* = 50,100
Age; missing values *n* = 0					
Age in years-median (IQR)	68.85 (65.95–75.04)	69.35 (65.98–75.69)	69.20 (65.95–75.42)	69.15 (65.88–75.19)	69.18 (65.96–75.46)
Sex; missing values *n* = 0					
Male	6394 (51.84)	10,437 (47.31)	5244 (40.76)	1034 (36.41)	23,109 (46.13)
Female	5940 (48.16)	11,623 (52.69)	7622 (59.24)	1806 (63.59)	26,991 (53.87)
Marital status; missing values *n* = 140 (0.28%)					
Living with partner	9852 (80.11)	17,180 (78.09)	10,117 (78.88)	2215 (78.13)	39,364 (78.79)
Living without partner	2446 (19.89)	4821 (21.91)	2709 (21.12)	620 (21.87)	10,596 (21.21)
Smoking status; missing values *n* = 614 (1.24%)					
Never	10,171 (84.50)	18,402 (84.31)	11,122 (86.94)	2475 (87.46)	42,170 (85.21)
Current or previous	1865 (15.50)	3425 (15.69)	1671 (13.06)	355 (12.54)	7316 (14.78)
Alcohol status; missing values *n* = 767 (1.55%)					
Never	11,224 (93.53)	20,243 (93.02)	11,903 (93.3)	2634 (93.40)	46,004 (93.25)
Occasionally	499 (4.16)	931 (4.28)	490 (3.84)	107 (3.79)	2027 (4.11)
Daily	278 (2.32)	589 (2.71)	356 (2.79)	79 (2.80)	1302 (2.64)
Physical activity; missing values *n* = 723 (1.46%)					
Never	8404 (69.78)	13,860 (63.71)	7678 (60.18)	1676 (59.41)	31,618 (64.03)
Occasionally	1085 (9.01)	2351 (10.81)	1434 (11.24)	306 (10.85)	5176 (10.48)
Daily	2554 (21.21)	5543 (25.48)	3647 (28.58)	839 (29.74)	12,583 (25.48)
BMI; missing values *n* = 1323 (2.71%)					
< 18.5	336 (2.82)	407 (1.90)	173 (1.37)	34 (1.22)	950 (1.95)
18.5–24.9	8091 (68.00)	11,943 (55.62)	5833 (46.24)	1215 (43.53)	27,082 (55.52)
25–29.9	3129 (26.30)	7612 (35.45)	5306 (42.06)	1187 (42.53)	17,234 (35.33)
30–34.9	324 (2.72)	1378 (6.42)	1175 (9.31)	316 (11.32)	3193 (6.55)
35–39.9	15 (0.13)	122 (0.57)	114 (0.90)	36 (1.29)	287 (0.59)
≥ 40	3 (0.03)	11 (0.05)	14 (0.11)	3 (0.11)	31 (0.06)
Chronic conditions					
Hypertension	0 (0.00)	17,057 (77.32)	12,162 (94.53)	2808 (98.87)	32,027 (63.93)
Diabetes	0 (0.00)	2242 (10.16)	6962 (54.11)	2516 (88.59)	11,720 (23.39)
CHD	0 (0.00)	2420 (10.97)	5714 (44.41)	2546 (89.65)	10,680 (21.32)
Stroke	0 (0.00)	77 (0.35)	424 (3.30)	425 (14.96)	926 (1.85)
COPD	0 (0.00)	157 (0.71)	352 (2.74)	266 (9.37)	775 (1.55)
Tumour	0 (0.00)	18 (0.08)	22 (0.17)	17 (0.60)	57 (0.11)
Mental disorders	0 (0.00)	79 (0.36)	93 (0.72)	81 (2.85)	253 (0.50)

*LTCs* Long-term conditions, *IQR* Interquartile range, *BMI* Body mass index, *CHD* Coronary heart disease, *COPD* Chronic obstructive pulmonary disease

The median follow-up was 4.72 years (IQR: 3.06–4.99). The all-cause mortality rates were substantially greater with the increase in LTC count. During 205,022.95 person-years of follow-up, the mortality rates (per 1000 person-years) were 15.92, 19.83, 21.18, and 24.80 in the no LTCs,1 LTC, 2 LTCs, and ≥ 3 LTCs groups, respectively. The number of LTCs at baseline had a significant association with all-cause mortality observed in the three models (Table 2). The adjusted ORs (95% CIs) for all-cause mortality in participants with 1, 2, and ≥ 3 LTCs compared with those with no LTCs were 1.45 (1.32–1.59), 1.72 (1.55–1.90), and 2.15 (1.85–2.50), respectively (*P*_trend_ < 0.001). We found that 10.44% in participants with all-cause mortality could be described as excessive incidence attributable to multimorbidity. In theory, we found that 299 cases with multimorbidity would need to be avoided in order to reduce the death for one person (Supplemental File 1: Table S1).

**Table 2:** Multimorbidity and all-cause mortality

	No LTCs	1 LTC	2 LTCs	≥3 LTCs	*P*_trend_
No. of deaths	760	1809	1146	297	
No. of person-years	47,741.92	91,206.57	54,098.89	11,975.57	
^a^Mortality rate	15.92	19.83	21.18	24.80	
		OR (95% CI)	OR (95% CI)	OR (95% CI)	
^b^Model1	Reference	1.35 (1.23,1.47)	1.56 (1.42,1.72)	1.96 (1.69,2.27)	< 0.001
^c^Model2	Reference	1.39 (1.27,1.53)	1.63 (1.47,1.80)	2.04 (1.76,2.36)	< 0.001
^d^Model3	Reference	1.45 (1.32,1.59)	1.72 (1.55,1.90)	2.15 (1.85,2.50)	< 0.001

*LTCs* Long-term conditions, *OR* denotes odds ratio and *95% CI* denotes 95% confidence interval

^a^Per 1000 person-years

^b^Model 1 is adjusted for age (< 75 or ≥ 75), sex (female or male) and marital status (living with partner or living without partner)

^c^Model 2 is adjusted for covariates in model 1 plus smoking status (never, current or previous), alcohol status (never, occasionally or daily) and physical activity (never, occasionally or daily)

^d^Model 3 is adjusted for covariates in model 2 plus body mass index (< 18.5, 18.5–24.9, 25–29.9, 30–34.9, 35–39.9 or ≥ 40)

In the study, we assessed the impact of 16 different disease clusters of 7 chronic diseases on the risk of all-cause mortality, as shown in Table 3. In the LTCs = 2 category, the cluster of hypertension + diabetes (OR 1.89, 95% CI 1.67–2.13), hypertension + CHD (OR 1.44, 95% CI 1.26–1.64), hypertension + stroke (OR 2.41, 95% CI 1.75–3.32), hypertension + COPD (OR 3.02, 95% CI 2.12–4.31), CHD + COPD (OR 2.02, 95% CI 1.03–3.95), and diabetes + COPD (OR 4.04, 95% CI 1.29–12.71) had significant effects on mortality. For those clusters with 3 diseases and 4 diseases, six different clusters had significant effects on all-cause mortality. Hypertension appears most frequently in disease clusters.

**Table 3:** The most impactful LTCs combinations in stratified logistic regression analysis for mortality, for different multimorbidity categories (based on LTC count)

Categories	No. of deaths	No. of person-years	Mortality rate^a^	Adjusted^b^ OR (95% CI)
2 LTCs (Total number of deaths *N* = 1146)				
Hypertension + diabetes	570	26,753.84	21.31	1.89 (1.67,2.13)
Hypertension + CHD	416	21,575.80	19.28	1.44 (1.26,1.64)
Hypertension + stroke	58	1581.35	36.68	2.41 (1.75,3.32)
Hypertension + COPD	44	992.24	44.34	3.02 (2.12,4.31)
Diabetes + CHD	28	2162.11	12.95	1.15 (0.77,1.72)
CHD + COPD	11	312.92	35.15	2.02 (1.03,3.95)
Hypertension + Mental disorders	8	284.27	28.14	2.08 (0.92,4.70)
Diabetes + COPD	4	89.62	44.63	4.04 (1.29,12.71)
3 LTCs (Total number of deaths *N* = 279)				
Hypertension + diabetes + CHD	188	8849.08	21.25	1.90 (1.59,2.27)
Hypertension + diabetes + stroke	29	660.92	43.88	3.48 (2.29,5.28)
Hypertension + stroke + CHD	17	652.11	26.07	2.09 (1.23,3.54)
Hypertension + CHD + COPD	17	477.75	35.58	2.57 (1.50,4.41)
Hypertension + diabetes + COPD	9	294.51	30.56	2.44 (1.16,5.11)
4 LTCs (Total number of deaths *N* = 18)				
Hypertension + diabetes +stroke + CHD	9	274.78	32.75	3.18 (1.52,6.65)

All predictors entered individually in separate models using No LTC group as the reference category

*LTCs* Long-term conditions, *CHD* Coronary heart disease, *COPD* Chronic obstructive pulmonary disease, *OR (95% CI)* Odds ratio and 95% confidence interval

^a^Per 1000 person-years; ^b^Adjusted for age, sex, marital status, smoking status, alcohol status, physical activity, and body mass index (< 18.5, 18.5–24.9, 25–29.9, 30–34.9, 35–39.9 or ≥ 40) at baseline

**Fig. 1:**
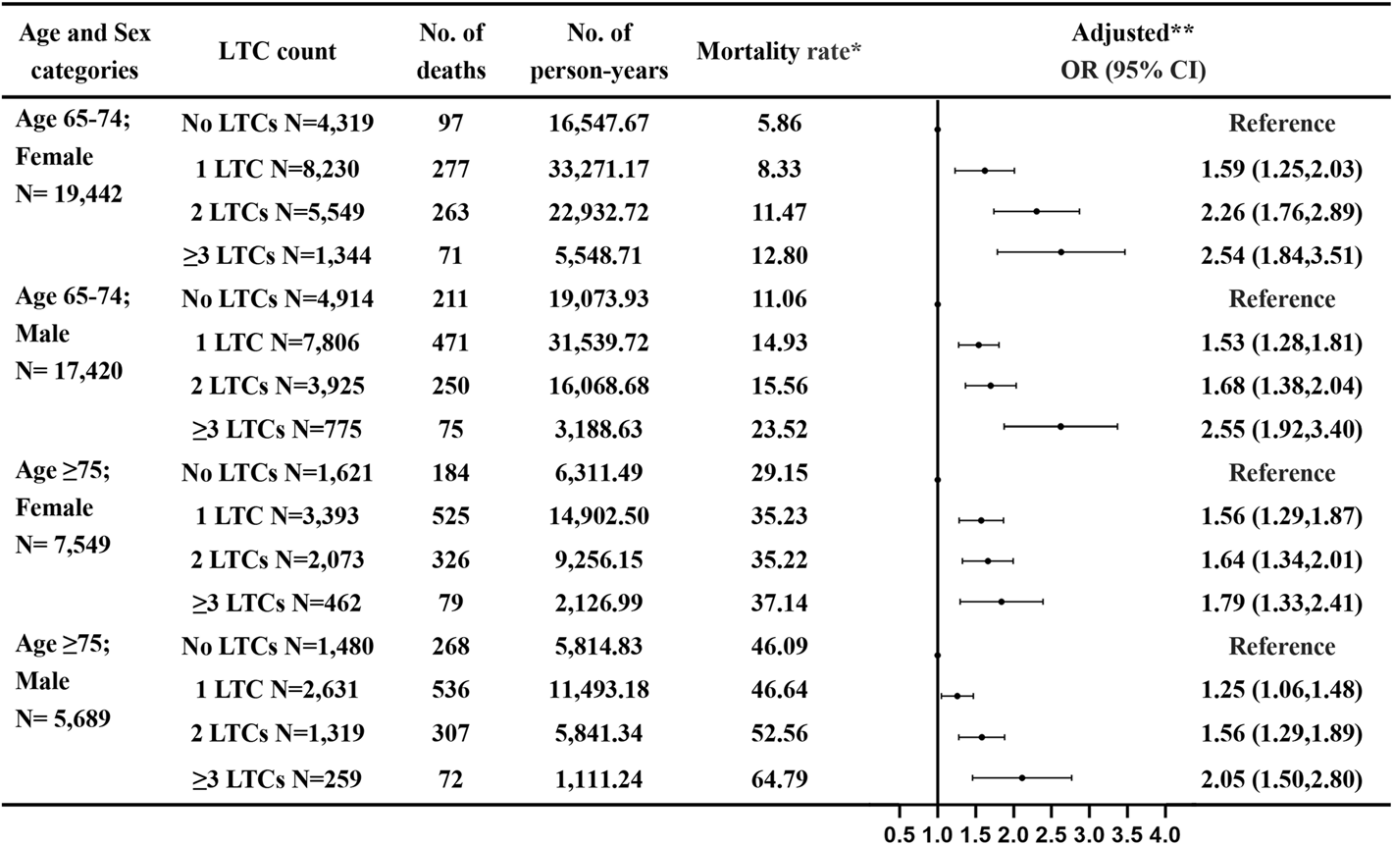
All-cause mortality for multimorbidity stratified by age and sex, and adjusted for marital status, smoking status, alcohol status, physical activity, and body mass index. One asterisk Per 1000 person-years; Two asterisks adjusted for marital status, smoking status, alcohol status, physical activity, and body mass index levels at baseline

The analysis of all-cause mortality for multimorbidity stratified by age and sex is shown in Fig. 1. In the multivariable-adjusted models, the absolute mortality rate was higher for the age group ≥75 years, but the relative risk for all-cause mortality were higher for the age group < 75 years. Furthermore, compared to participants with no LTCs in the age group < 75 years, participants with ≥3 LTCs had the highest relative risk of all-cause mortality (OR 2.55, 95% CI 1.92–3.40 for males and OR 2.54, 95% CI 1.84–3.51 for females). The observed mortality risk was similar for both female and male in the same age group. In addition, we found that being older, living without partner, and being underweight had a higher risk of mortality. In contrast, participants who were female, overweight, class I obesity, and physical activity had a significantly lower adjusted risk of all-cause mortality (Supplemental File 1: Table S2). The results of sensitivity analysis yielded similar findings as our main results: as compared with no LTCs, the risk of all-cause mortality increased with increasing number of LTCs. (Supplemental File 1: Table S3, Table S4 and Table S5).

## Discussion

This cohort study demonstrated a positive association of multimorbidity with all-cause mortality. The results from sensitivity analyses were robust. In the LTCs ≥2 category, the chronic disease cluster that included hypertension, diabetes, CHD, COPD, and stroke had significant effects on all-cause mortality. In addition, we found a higher absolute mortality rate among those ≥75 years, but the relative magnitude of the effect on mortality risk was greater among those < 75 years.

The higher all-cause mortality risk among participants with 1, 2, and ≥ 3 LTCs found in this study is consistent with previous studies that have focused on older adults [30, 31]. The study of *Martinez-Gomez* et al. observed significant upward trends with 1 LTC (HR 1.26), 2 LTC (HR 1.78), and ≥ 3 LTC (HR 2.27) than those without LTC [30]. Similarly, similar results were observed by *Nunes* et al. [10]. And we also found that disease clusters were most strongly associated with all-cause mortality relative to the number of multimorbidity alone, whereas previous studies mainly focused on the role of a single disease in death, such as diabetes [32]. Moreover, the present study found that hypertension was the most frequent occurrence, with presence in 11 of the 16 different disease clusters, suggesting that there may be a potential link between hypertension and a variety of chronic diseases. However, a large sample of participants aged 40 to 69 years study based on 36 chronic conditions showed that conditions such as hypertension, diabetes, and asthma were at the center of common multimorbidity [16]. These findings suggest there is a need for more research to explore the links between chronic diseases and their possible interactions, which is of great significance for the guidance and treatment of multimorbidity, and also provides a theoretical basis for the formulation of health management measures and allocation of medical resources for the elderly population to some extent.

A prospective population-based cohort study of England people aged 37 to 73 years shown that participants aged 37–49 years with ≥4 LTCs had the highest relative risk of all-cause mortality [11]. And we found that the association on risk of all-cause mortality with an increasing number of LTCs was particularly evident among younger age groups (< 75 vs ≥ 75 years old), which not only fills in the gap of the age of participants, but also is basically consistent with the trend of previous studies. This study found that female had a higher prevalence of multimorbidity than male, which was consistent with previous findings [33, 34]. However, the observed magnitude of mortality effect size was similar for both female and male. Potential explanations for the phenomenon are that females are generally more sensitized to their health [34], more likely to report more conditions [35], and more likely to engage in preventive health behavior [36]. These findings suggest the need for early intervention to manage and prevent chronic diseases and to reduce the prevalence of multiple diseases as much as possible.

Studies have shown that the causes of death from multimorbidity can be attributed to 4 major underlying risk factors: smoking, alcohol consumption, underweight, and physical inactivity [11, 37]. While previous studies found that smoking was a risk factor for all-cause mortality [11], our results found no statistically significant difference, which is possibly explained by the quitter bias (people may stop smoking because they are in poor health and may be advised not to continue). The results of the risk study on alcohol consumption were basically consistent with the results of *Ortolá* et al., which showed that there was no statistically significant difference in mortality between light-to-moderate alcohol consumption and no alcohol consumption among people over 60 years old [38]. As well, we found the greater all-cause mortality risk among persons with underweight (BMI < 18.5 kg/m^2^) in this study, which agreed with previous studies [21, 39, 40]. In addition, this study suggests that physical activity is associated with a lower mortality risk, and the underlying mechanism behind this finding may be that physical activity delays disease progression by preventing many chronic diseases, including diabetes, cardiovascular and respiratory diseases, and some types of cancer [41]. Therefore, once any of these chronic conditions is diagnosed, physical activity is often incorporated into treatment plans to improve quality of life and survival [30, 41, 42].

Our study has several strengths. First, the determination of chronic diseases was relatively accurate and comprehensive, including the self-reported condition of participants and the diagnosis made by the physician based on the professional comprehensive examination. Second, our study was also novel in that we explored disease clusters that were associated with the highest risk of death. In addition, it is more convincing to assess the relationship between multimorbidity and all-cause mortality based on a large sample size. Finally, the main results remained robust after conducting sensitivity analyses. However, the study has some limitations. Since we included only seven chronic diseases registered at baseline, some other diseases associated with older people such as hyperlipidemia and arthritis could not be taken into account, which may underestimate the prevalence of many diseases in this study, and there was no way to estimate the severity of chronic diseases as well. Another restriction is that our participants were people over 65 years, which should caution generalizing our findings to younger age groups. In addition, although we adjusted for various covariates, there is still a possibility of residual confounding, such as diet factors. Last, recall bias is unavoidable in self-reported information.

## Conclusion

Our findings suggest an increased risk of all-cause mortality with an elevated number of multimorbidity, particularly disease clusters, which provides scientific basis for the treatment, prevention and control of the multimorbidity, and has important public health significance in guiding the rational allocation of medical and health resources.

## Supplementary Information

**Additional file 1: Supplementary Figure 1.** Flow diagram of participant selection. **Supplemental Table 1.** The population attributable risk percent and the number needed to screened for all-cause mortality based on LTC count. **Supplemental Table 2.** Multimorbidity and all-cause mortality: Multiple logistic regression analysis. This table shows that being older, living without partner, and being underweight had a higher risk of mortality. In contrast, participants who were female, overweight, class I obesity, and physically active had a significantly lower adjusted risk of all-cause mortality. **Supplemental Table 3.** Comparison of LTCs (self-report vs physical examination) in prediction of all-cause mortality. **Supplemental Table 4.** Multivariable Adjusted odds ratio (95% confidence interval) for multimorbidity and all-cause mortality according to the different classification by BMI. **Supplemental Table 5.** The most impactful LTCs combinations in stratified logistic regression analysis for mortality (based on LTC count). these three tables show that the sensitivity analysis yielded similar findings as our main results, and the risk of death increased with the increase in LTC count.

## Data Availability

The datasets used and/or analyzed in this study were obtained from third parties and cannot be publicly available to confidentiality requirements.

## Abbreviations

LTCs: Long-term conditions
FPG: Fasting plasma glucose
SBP: Systolic blood pressure
DBP: Diastolic blood pressure
BMI: Body mass index
CHD: Coronary heart disease
COPD: Chronic obstructive pulmonary disease
IQR: Interquartile range
ORs: Odds ratios
CIs: Confidence intervals
PAR%: The population attributable risk percent
NNBS: The number needed to screened
