# Supplementary material for: Relationship between multimorbidity, disease cluster and all-cause mortality among older adults: a retrospective cohort analysis
Source: BMC Public Health. 2021 Jun 5;21:1080. doi: 10.1186/s12889-021-11108-w (PMC8180153; doi:10.1186/s12889-021-11108-w)
Supplement: Supplementary file 1 — Additional file 1: Supplementary Figure 1. Flow diagram of participant selection. Supplemental Table 1. The population attributable risk percent and the number needed to screened for all-cause mortality based on LTC count. Supplemental Table 2. Multimorbidity and all-cause mortality: Multiple logistic regression analysis. This table shows that being older, living without partner, and being underweight had a higher risk of mortality. In contrast, participants who were female, overweight, class I obesity, and physically active had a significantly lower adjusted risk of all-cause mortality. Supplemental Table 3. Comparison of LTCs (self-report vs physical examination) in prediction of all-cause mortality. Supplemental Table 4. Multivariable Adjusted odds ratio (95% confidence interval) for multimorbidity and all-cause mortality according to the different classification by BMI. Supplemental Table 5. The most impactful LTCs combinations in stratified logistic regression analysis for mortality (based on LTC count). these three tables show that the sensitivity analysis yielded similar findings as our main results, and the risk of death increased with the increase in LTC count. [file 12889_2021_11108_MOESM1_ESM.docx]

**Supplemental Figure**


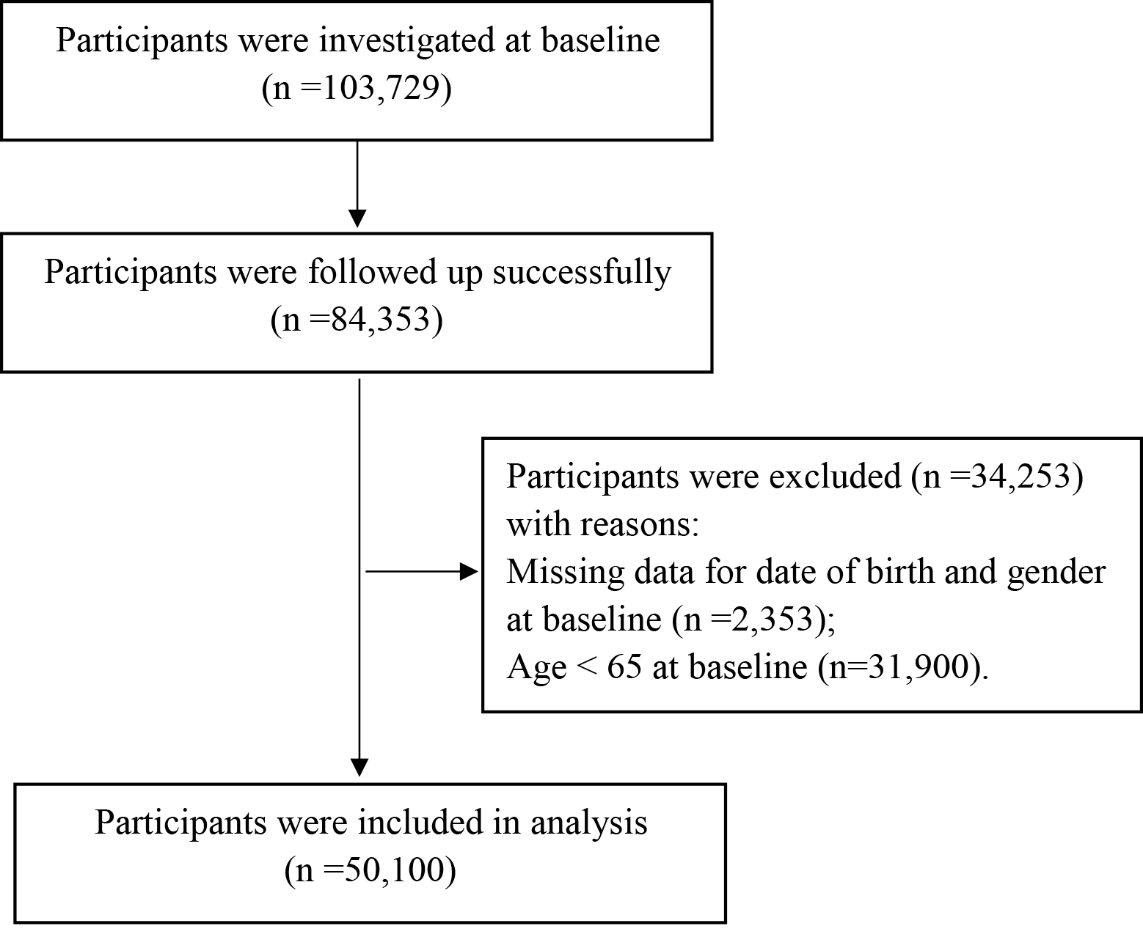


**Figure S1 Flow diagram of participant selection**

**Supplemental Tables**

**Table S1** The population attributable risk percent and the number needed to screened for all-cause mortality based on LTC count.

|  | No. of deaths | No. of person-years | Mortality rate* | PAR% | NNBS |
| --- | --- | --- | --- | --- | --- |
| No (<2 LTC) | 2,569 | 138,948.49 | 18.49 | 10.44 | 299 |
| Yes (≥2 LTC) | 1,443 | 66,074.46 | 21.84 |  |  |
| Total | 4,012 | 205,022.95 | 19.56 |  |  |

LTCs, long-term conditions; One asterisk Per 1,000 person-years; PAR%, the population attributable risk percent; NNBS, the number needed to screened.

**Table S2** Multimorbidity and all-cause mortality: Multiple logistic regression analysis.

| Predictor variables | Categories for predictor variables | Adjusted* OR(95% CI) |
| --- | --- | --- |
| LTC categories | No LTCs (reference) | 1 |
|  | 1 LTC | 1.45 (1.32,1.59) |
|  | 2 LTC | 1.72 (1.55,1.90) |
|  | ≥3 LTC | 2.15 (1.85,2.50) |
| Age | <75(reference) | 1 |
|  | ≥75 | 3.93 (3.66,4.22) |
| Sex | Male (reference) | 1 |
|  | Female | 0.57 (0.53-0.62) |
| Marital status | Living with partner (reference) | 1 |
|  | Living without partner | 1.41(1.31,1.53) |
| Smoking status | Never Smoked (reference) | 1 |
|  | Current or Previous Smoker | 0.99 (0.88-1.10) |
| Physical activity | Never (reference) | 1 |
|  | Occasionally | 0.92 (0.82-1.03) |
|  | Daily | 0.78 (0.71-0.84) |
| Alcohol status | Never (reference) | 1 |
|  | Occasionally | 0.84 (0.69-1.01) |
|  | Daily | 0.88 (0.70-1.11) |
| Body mass index | 18.5-25 (reference) | 1 |
|  | <18.5 | 1.47 (1.21-1.78) |
|  | 25-30 | 0.76 (0.71-0.83) |
|  | 30-35 | 0.80 (0.69-0.94) |
|  | 35-40 | 1.04 (0.68-1.60) |
|  | >40 | 0.79 (0.23-2.72) |

One asterisk adjusted for age, sex, marital status, smoking status, alcohol status, physical activity, and body mass index at baseline.

**Table S3** Comparison of LTCs (self-report vs physical examination) in prediction of all-cause mortality.

|  | LTC count | No. of deaths | No. of person-years | Mortality rate* | Adjusted**  OR (95% CI) |
| --- | --- | --- | --- | --- | --- |
| self-report N= 50,100 | No LTCs N=20,705 | 1,387 | 80,557.82 | 17.22 | Reference |
|  | 1 LTC N=20,307 | 1,761 | 85,608.54 | 20.57 | 1.34 (1.11,1.61) |
|  | 2 LTCs N=8,074 | 751 | 34,455.02 | 21.80 | 1.73 (1.40,2.14) |
|  | ≥3 LTCs N=1,014 | 113 | 4,398.64 | 25.69 | 2.28 (1.64,3.17) |
| physical examination N= 50,100 | No LTCs N=17,188 | 1,101 | 68,607.63 | 16.05 | Reference |
|  | 1 LTC N=22,126 | 1,894 | 91,747.43 | 20.64 | 1.45 (1.32,1.59) |
|  | 2 LTCs N=9,527 | 888 | 39,529.25 | 22.46 | 1.72 (1.55,1.90) |
|  | ≥3 LTCs N=1,259 | 129 | 5,138.65 | 25.10 | 2.15 (1.85,2.50) |

LTCs, long-term conditions; OR (95% CI), odds ratio and 95% confidence interval.

One asterisk Per 1,000 person-years; Two asterisks adjusted for age, sex, marital status, smoking status, alcohol status, physical activity, and body mass index (<18.5, 18.5-24.9, 25-29.9, 30-34.9, 35-39.9 or ≥40) at baseline.

**Table S4** Multivariable Adjusted odds ratio (95% confidence interval) for multimorbidity and all-cause mortality according to the different classification by BMI.

|  | No LTCs | 1 LTC | 2 LTCs | ≥3 LTCs | *P* _trend_ |
| --- | --- | --- | --- | --- | --- |
| No. of deaths | 760 | 1,809 | 1,146 | 297 |  |
| No. of person-years | 47,741.92 | 91,206.57 | 54,098.89 | 11,975.57 |  |
| ^a^Mortality rate | 15.92 | 19.83 | 21.18 | 24.80 |  |
| Multivariable adjusted* | Reference | 1.45 (1.32,1.59) | 1.72 (1.55,1.90) | 2.15 (1.85,2.50) | <0.001 |
| Multivariable adjusted** | Reference | 1.46 (1.33,1.60) | 1.75 (1.58,1.93) | 2.22 (1.92,2.58) | <0.001 |

LTCs, long-term conditions;

^a^ Per 1,000 person-years;

*Multivariable adjusted for age, sex, marital status, smoking status, alcohol status, physical activity and body mass index (<18.5, 18.5-24.9, 25-29.9, 30-34.9, 35-39.9 or ≥40);

**Multivariable adjusted for age, sex, marital status, smoking status, alcohol status, physical activity and body mass index (<18.5, 18.5-22.9, 23-24.9, 25-29.9, or ≥30).

**Table S5** The most impactful LTCs combinations in stratified logistic regression analysis for mortality (based on LTC count).

| Categories | Adjusted*  OR (95% CI) | Adjusted**  OR (95% CI) |
| --- | --- | --- |
| 2 LTCs (Total number of deaths N = 1146) |  |  |
| Hypertension + diabetes | 1.89 (1.67,2.13) | 1.93 (1.71,2.18) |
| Hypertension + CHD | 1.44 (1.26,1.64) | 1.45 (1.27,1.65) |
| Hypertension + stroke | 2.41 (1.75,3.32) | 2.59 (1.89,3.54) |
| Hypertension + COPD | 3.02 (2.12,4.31) | 3.04 (2.14,4.33) |
| Diabetes + CHD | 1.15 (0.77,1.72) | 1.15 (0.77,1.72) |
| CHD + COPD | 2.02 (1.03,3.95) | 1.97 (1.01,3.85) |
| Hypertension + Mental disorders | 2.08 (0.92,4.70) | 2.07 (0.92,4.68) |
| Diabetes + COPD | 4.04 (1.29,12.71) | 3.86 (1.23,12.07) |
| 3 LTCs (Total number of deaths N = 279) |  |  |
| Hypertension + diabetes + CHD | 1.90 (1.59,2.27) | 1.95 (1.64,2.33) |
| Hypertension + diabetes + stroke | 3.48 (2.29,5.28) | 3.72 (2.47,5.61) |
| Hypertension + stroke + CHD | 2.09 (1.23,3.54) | 2.14 (1.26,3.62) |
| Hypertension + CHD + COPD | 2.57 (1.50,4.41) | 2.51 (1.46,4.31) |
| Hypertension + diabetes + COPD | 2.44 (1.16,5.11) | 2.40 (1.52,5.01) |
| 4 LTCs (Total number of deaths N = 18) |  |  |
| Hypertension + diabetes +stroke + CHD | 3.18 (1.52,6.65) | 3.23 (1.54,6.76) |

All predictors entered individually in separate models using No LTC group as the reference category.

LTCs, long-term conditions; CHD, coronary heart disease; COPD, chronic obstructive pulmonary disease; OR (95% CI), odds ratio and 95% confidence interval.

One asterisk adjusted for age, sex, marital status, smoking status, alcohol status, physical activity and body mass index (<18.5, 18.5-24.9, 25-29.9, 30-34.9, 35-39.9 or ≥40);

Two asterisks adjusted for age, sex, marital status, smoking status, alcohol status, physical activity and body mass index (<18.5, 18.5-22.9, 23-24.9, 25-29.9, or ≥30).
